# Identifying Insects with Incomplete DNA Barcode Libraries, African Fruit Flies (Diptera: Tephritidae) as a Test Case

**DOI:** 10.1371/journal.pone.0031581

**Published:** 2012-02-16

**Authors:** Massimiliano Virgilio, Kurt Jordaens, Floris C. Breman, Thierry Backeljau, Marc De Meyer

**Affiliations:** 1 Royal Museum for Central Africa, Tervuren, Belgium; 2 Royal Belgian Institute of Natural Sciences, Brussels, Belgium; 3 Joint Experimental Molecular Unit, Tervuren, Belgium; 4 Department of Biology, University of Antwerp, Antwerp, Belgium; Biodiversity Insitute of Ontario - University of Guelph, Canada

## Abstract

We propose a general working strategy to deal with incomplete reference libraries in the DNA barcoding identification of species. Considering that (1) queries with a large genetic distance with their best DNA barcode match are more likely to be misidentified and (2) imposing a distance threshold profitably reduces identification errors, we modelled relationships between identification performances and distance thresholds in four DNA barcode libraries of Diptera (n = 4270), Lepidoptera (n = 7577), Hymenoptera (n = 2067) and Tephritidae (n = 602 DNA barcodes). In all cases, more restrictive distance thresholds produced a gradual increase in the proportion of true negatives, a gradual decrease of false positives and more abrupt variations in the proportions of true positives and false negatives. More restrictive distance thresholds improved precision, yet negatively affected accuracy due to the higher proportions of queries discarded (*viz.* having a distance query-best match above the threshold). Using a simple linear regression we calculated an *ad hoc* distance threshold for the tephritid library producing an estimated relative identification error <0.05. According to the expectations, when we used this threshold for the identification of 188 independently collected tephritids, less than 5% of queries with a distance query-best match below the threshold were misidentified. *Ad hoc* thresholds can be calculated for each particular reference library of DNA barcodes and should be used as cut-off mark defining whether we can proceed identifying the query with a known estimated error probability (*e.g.* 5%) or whether we should discard the query and consider alternative/complementary identification methods.

## Introduction

DNA barcoding refers to the identification of a species by (1) calculating the genetic distances between the COI sequence of an unknown specimen ( = a query) and a collection of well-identified reference COI sequences in a library ( = DNA barcodes) and (2) assigning to the query the species name of the reference sequence with the smallest genetic distance [1]. Critical for DNA barcoding identification is either the availability of such libraries of well identified referenced DNA barcodes and the degree of taxonomic coverage of these libraries [2], [3]. So far, comprehensive barcode libraries only exist for a number of relatively well-known taxonomic groups (*e.g.* [4], [5], [6], [7]) or for limited geographic ranges (*e.g.* [8], [9]). Yet for most taxa reference libraries are still largely incomplete, in terms of both species and population coverages, so that they cannot yet be used for reliable identification [2], [10]. In practice, whenever a query is not represented by a conspecific DNA barcode in the reference library, it will be erroneously assigned to the most similar heterospecific DNA barcode in the library. Hence, queries showing high genetic distances with their best match may indicate that there are no conspecific DNA barcodes for that query in the reference library. The extent of this problem can be reduced, however, by defining distance thresholds so that a query is discarded (*i.e.* its identification is considered unreliable) whenever the distance between the query and its best DNA barcode match exceeds the threshold value.

Currently, several types of distance threshold can be implemented. The Barcode of Life system uses a fixed 1% distance threshold [1], while fixed 2% or 3% thresholds were common in earlier barcoding studies [11]. A more flexible approach is provided by the so-called DNA barcoding gap, *i.e.* the degree of separation between intraspecific and congeneric-interspecific distance distributions. Hebert *et al.* [4] proposed to use a distance value corresponding to 10 times the average intraspecific variation (the 10× threshold) as a screen for new species. This threshold has been considered in DNA barcoding as a tool to discriminate between con- and hetero-specific identifications. Unfortunately no single interspecific distance threshold applies to all taxonomic groups since patterns of intra- *vs.* interspecific sequence divergence vary across taxa. For example, Meyer and Paulay [2] showed that thresholds of 3.2× to 6.8× were more suitable for the identification of marine gastropods and still alternative distance thresholds have been proposed for other taxa (*e.g.* [12]). Yet, recent evidence suggests that the barcoding gap and its distance thresholds may not be good predictors of the identification success of DNA barcoding [10], [11], [13], [14], [15]. Meier *et al.* [14] proposed to adapt distance thresholds to the particular reference library used. To this end they suggested to use a threshold corresponding to the 95^th^ percentile of the intraspecific- distance distribution calculated from the reference library. One of the advantages of this method is that the threshold can be easily recalculated every time the library is updated with new reference DNA barcodes.

Of course, a suitable distance threshold is just one of several factors to consider in the DNA barcoding identification of species. Among others, it is also of tantamount importance that the library properly represents the taxonomic diversity of the group to be identified. Taxon coverage is a particularly important issue in insects, with their more than one million described species and their probably several millions of still undescribed species [16]. As such, Meier *et al.* [13], [14] showed that identification success of DNA barcoding in Diptera is relatively low (<70%) but they did not distinguish between different error sources. Virgilio *et al.* [15] investigated relationships between identification success and taxon coverage in insects and suggested that the combined effects of false positives (*i.e.* producing an erroneous positive identification) and false negatives (*i.e.* erroneously discarding a query) can heavily affect the reliability of insect DNA barcoding. In view of the limits of identifying species when using libraries with incomplete taxon coverage, it would be useful to develop working strategies by which the extensive DNA barcode libraries that are already available on the Barcode of Life Data Systems (BOLD, http://www.boldsystems.org) can be profitably used. In this context, the Consortium for the Barcoding of Life (http://www.barcoding.si.edu) initiated, amongst others, the Tephritid Barcoding Initiative (TBI) as a demonstrator project whose main aim is to develop a DNA barcoding system for the identification of fruit flies (Diptera: Tephritidae).

Tephritid fruit flies, or “true” fruit flies, are a family of main economic significance including nearly 5000 species worldwide. Several species are pests of (sub)tropical horticultural crops and pose a major threat to production and international trade of crops. Therefore, National Plant Protection Organizations (NPPOs) are pressed to have a working strategy that allows fast and correct identifications without some of the current limitations of morphological taxonomy (including the decreasing number of morphological taxonomists and the difficulty of identifying immature stages).

The present contribution aims at proposing a protocol for the identification of species through DNA barcoding. Starting from the observation that queries showing large sequence divergence with their best-matched reference DNA barcodes are more likely to be misidentified, we modeled relationships between distance thresholds and identification performance and used tephritid fruit flies from a particular geographic region (Afrotropics) as a test case. The objectives of this work were to (1) verify the generality of relationships between performances of DNA barcoding and distance thresholds in different insect reference libraries, (2) assess changes in the identification success for libraries with different levels of taxon coverage (3) assemble a reference DNA barcode library for African frugivorous tephritids, (4) calculate a theoretical distance threshold producing an estimated identification error <5% with respect to the identification of those tephritids, (5) verify if the theoretical distance threshold and the tephritid reference library can be reliably used to identify unknown samples of tephritids sourced from quarantine interceptions and surveys, and (6) propose a general protocol for the identification of species when using incomplete DNA barcode libraries.

## Methods

Three large insect libraries including (1) 4270 barcodes of Diptera, (2) 7577 barcodes of Lepidoptera and (3) 2067 barcodes of Hymenoptera (from 345, 1168 and 160 species, respectively) were assembled by using the sequence alignment provided in Virgilio *et al.* [15]. Sequence list, collection methods, alignment procedures and taxonomic composition of libraries are detailed in [15]. In addition, we built a regional tephritid reference library by including 602 DNA barcodes from indigenous African tephritids and fruit fly species alien to Africa. Emphasis was given to the three main fruit fly genera of economic relevance in Africa *viz. Ceratitis*, *Dacus* and *Bactrocera* (Data S1). DNA extraction, amplification and sequencing were performed following methods and protocols described in Virgilio *et al.* [17] and Van Houdt *et al.* [18]. The reference tephritid specimens were deposited in the collections of the Royal Museum for Central Africa (Tervuren, BE). All DNA sequences considered in this study included at least 550 bp and were aligned and trimmed in order to include only the mtDNA COI barcode region, *i.e.* the 658 bp COI fragment amplified by the “universal primers” of Folmer *et al.* [19].

For each of the four libraries we simulated DNA barcoding identification by using each sequence in a library as a query against all the other barcodes of that library. Identifications were based on Kimura's two parameter (K2P) genetic distance [20] as it is the most commonly used and widely accepted distance metric in DNA barcoding (but see [21]). SpeciesIdentifier v1.5 [14] was used to calculate pairwise Kimura's two parameter (K2P) distances [20] and to quantify the proportion of correctly identified queries according to two distance based identification criteria: Best Match (BM) and Best Close Match (BCM). With BM, each query is simply assigned the species name of the most similar DNA barcode (smallest K2P genetic distance). With BCM a distance threshold is introduced such that only queries whose K2P distance to their most similar (BM) barcode remains below the threshold are considered as correctly identified while queries with K2P distance above the threshold are discarded. Queries that produced identical K2P distances with DNA barcodes from multiple species were considered as misidentified. The K2P distance threshold of BCM allows subdividing query results into (1) true positives (TP), *i.e.* queries that are correctly identified with a K2P distance to their best match below the threshold, (2) false positives (FP), *viz.* queries that are misidentified despite the K2P distance to their best match remains below the threshold, (3) true negatives (TN), misidentified queries that are correctly rejected because the K2P distance to their best match is above the threshold and (4) false negatives (FN), correctly identified queries that are erroneously discarded as their K2P distance to their best match is above the threshold. The performances of BCM were quantified by calculating accuracy ((TP+TN)/total number of queries) and precision (TP/(number of not discarded queries)). We also quantified the overall identification (ID) error ((FP+FN)/total number of queries) and the relative ID error (FP/number of not discarded queries). The former corresponds to the overall proportion of misidentified queries, the latter to the proportion of misidentified queries that were not discarded (note that overall ID error = 1-accuracy and relative ID error = 1-precision and that the maximum value accuracy and precision can reach is 1). Variations in the proportions of TP, TN, FP, FN, accuracy, precision, overall and relative ID errors were quantified for 30 arbitrary K2P distance thresholds (THR_K2P_) ranging from THR_K2P_ = the largest query-best match K2P distance in a library (all queries are accepted as being correctly identified, *i.e.* none is discarded as in the BM criterion) to THR_K2P_ = 0.00 (only identical sequences are accepted as being correctly identified, all the others are discarded). Relationships between relative ID errors and K2P distance thresholds were investigated in each of the four libraries through linear regression. Model parameters, 95% confidence intervals and goodness of fit were calculated in OriginPro v7 (http://www.originlab.com). For each library, the regression equation was used to infer an *ad hoc* distance threshold corresponding to the K2P distance yielding a relative ID error <0.05 (THR_K2P_0.05_). This *ad hoc* threshold corresponds to the K2P distance at which 95% of the not discarded queries are expected to be correctly identified.

Relationships between levels of taxon coverage of a DNA barcode reference library, K2P distance thresholds and the proportions of overall and relative ID errors were investigated through simulations. Unlike the tephritid regional library, the Diptera, Lepidoptera and Hymenoptera libraries were built by including at least two DNA barcodes for each species (see [15]). We used these three libraries to simulate a reference library with 100% taxon coverage, *i.e.* in which each query had at least one conspecific DNA barcode in the library. We then reduced levels of taxon coverage in the libraries by randomly eliminating all barcodes but one in 25%, 50% and 75% of the species in each library. These reduced datasets thus simulated libraries with 75%, 50% and 25% of taxon coverage (as all species for which only single sequences were left in the library no longer had a conspecific reference DNA barcode when that single sequence was used for a query). Each randomization was repeated three times and for each simulation we calculated overall and relative ID errors and estimated THR_K2P_0.05_ following the methods described above.

To test the reliability of the DNA barcoding identification in a “real life scenario”, we considered an independent set of 188 adult tephritids intercepted by European National Plant Protection Organizations (NPPOs) or sampled during recent monitoring surveys in Africa (including Togo, DR Congo and Mozambique). These tephritid interceptions were identified to species level using morphological characters and then blind tested by generating a barcode sequence (methods according to [17], [18]) that was compared with the aforementioned regional tephritid library. In this way we verified whether the THR_K2P_0.05_ threshold calculated from the tephritid reference library could yield a relative ID error <0.05 in a newly generated set of tephritid specimens.

## Results

**Figure 1:**
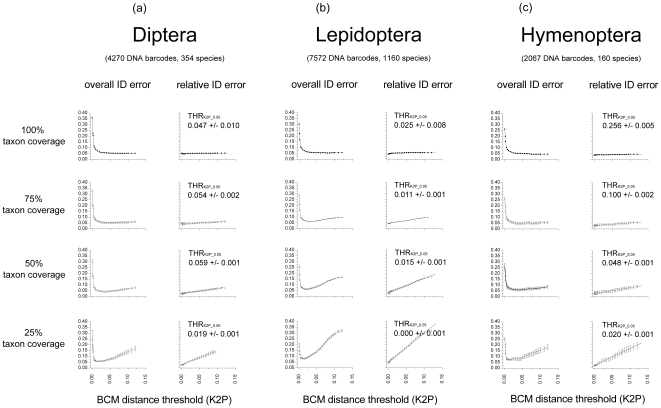
a, b, c: Relationships between ID errors and taxon coverage of libraries. Overall ID errors ((FP+FN)/total number of queries) and relative ID errors (FP/number of not discarded queries) at 30 arbitrary BCM distance thresholds in a) Diptera, b) Lepidoptera and c) Hymenoptera. In the 100% taxon coverage simulation, each query had at least a conspecific in the reference database. In the remaining simulations 25%, 50% and 75% of query species were not represented in the reference library (corresponding to 75%, 50% and 25% of taxon coverage). Simulations of 75%, 50% and 25% taxon coverage were repeated three times (standard errors as error bars). For each simulation, *ad hoc* distance thresholds (THR_K2P_0.05_) corresponding to a relative ID error <0.05 were inferred from linear fitting.

Simulations on the three large libraries of Diptera, Lepidoptera and Hymenoptera yielded remarkably consistent patterns of variation of TP, FP, TN, FN, accuracy and precision (Data S4, S5, S6). In all cases, more restrictive BCM distance thresholds produced a gradual increase of TN, a gradual decrease in FP and more abrupt variations in the proportions of TP (decreasing) and FN (increasing). With these libraries, the use of more restrictive thresholds also resulted in a marked drop of accuracy and in a gradual improvement of precision. BM identification performances in the large insect libraries (simulating100% taxon coverage) were generally better than those of the tephritid library, yet the variations in precision when using more restrictive BCM thresholds were less pronounced. When passing from no threshold (BM) to the most restrictive threshold value (THR_K2P_ = 0.00) precision increased in Diptera with only 0.7% (from 0.946 to 0.953), in Lepidoptera with 1.4% (from 0.944 to 0.958) and in Hymenoptera with 1% (from 0.955 to 0.966). Accordingly, the use of more restrictive BCM thresholds reduced the relative ID error from 0.054 to 0.047 in Diptera, from 0.056 to 0.042 in Lepidoptera and from 0.045 to 0.034 in Hymenoptera. Hence in the Hymenoptera library, the BM criterion could already produce a relative ID error <0.05. THR_K2P_0.05_ values in libraries simulating different levels of taxon coverage (Table 1) ranged from 0.019 (+/−0.001) to 0.059 (+/−0.002) in Diptera, from 0.000 (+/−0.001) to 0.025 (+/−0.008) in Lepidoptera and from 0.020 (+/−0.001) to 0.256 (+/−0.005) in Hymenoptera. Regardless of variability observed among THR_K2P_0.05_ values, relationships between relative ID error estimates and slope of the linear fitting y = a+bx were consistent across insect orders with higher slope values in libraries with lower taxon coverage. In Diptera the slope resulting from the 25% taxon coverage simulation was 34.6 times larger than the value obtained from the 100% taxon coverage simulation, similarly in Lepidoptera and Hymenoptera it was 28.9 and 30.4 times larger (Table 1, Fig. 1a, b, c).

**Table 1:** Relationships between levels of taxon coverage and relative ID error.

y = a+bx		Diptera				Lepidoptera				Hymenoptera			
				t-value				t-value				t-value	
**100%% taxon coverage**	a (intercept)	0.048	+/−0.000	140.911	***	0.048	+/−0.001	67.125	***	0.037	+/−0.000	141.030	***
	b (slope)	0.036	+/−0.006	6.486	***	0.087	+/−0.012	7.515	***	0.049	+/−0.004	11.333	***
	THR_K2P_0.05_	0.047	+/−0.009			0.025	+/−0.008			0.256	+/−0.005		
	F_1,25_	42.062			***	56.477			***	128.446			***
	R	0.798				0.838				0.918			
**75%% taxon coverage**	a (intercept)	0.042	+/−0.000	127.837	***	0.045	+/−0.001	78.312	***	0.028	+/−0.000	57.313	***
	b (slope)	0.158	+/−0.005	29.958	***	0.484	+/−0.009	52.327	***	0.220	+/−0.008	27.730	***
	THR_K2P_0.05_	0.054	+/−0.002			0.011	+/−0.001			0.100	+/−0.002		
	F_1,25_	897.488			***	2738.131			***	768.943			***
	R	0.987				0.996				0.985			
**50%% taxon coverage**	a (intercept)	0.024	+/−0.000	58.413	***	0.032	+/−0.001	23.510	***	0.026	+/−0.001	37.885	***
	b (slope)	0.44	+/−0.007	65.388	***	1.222	+/−0.022	56.092	***	0.500	+/−0.011	44.859	***
	THR_K2P_0.05_	0.059	+/−0.001			0.015	+/−0.001			0.048	+/−0.001		
	F_1,25_	4275.572			***	3146.289			***	2012.285			***
	R =	0.997				0.996				0.994			
**25%% taxon coverage**	a (intercept)	0.026	+/−0.001	27.043	***	0.050	+/−0.003	17.559	***	0.020	+/−0.002	10.426	***
	b (slope)	1.245	+/−0.016	79.005	***	2.516	+/−0.046	54.719	***	1.491	+/−0.031	48.372	***
	THR_K2P_0.05_	0.019	+/−0.001			0.000	+/−0.001			0.020	+/−0.001		
	F_1,25_	6241.857			***	2994.223			***	2339.885			***
	R	0.998				0.996				0.995			

Regression (see Figures 3a, b, c) was fitted according to the linear model y = a+bx and THR_K2P_0.05_ (*i.e.* the *ad hoc* threshold corresponding to a relative ID error <0.05) calculated. Goodness-of-fit is indicated by values of R close to 1.00 and by the statistical significance of (1) F-test of the overall fit (1 and 25 degrees of freedom), (2) t-tests of individual parameters.

***: p<0.001, regression 95% confidence intervals are indicated.

The regional library of tephritid DNA barcodes (Data S1) comprised 153 frugivorous species of the following genera: *Bactrocera* (9 species, 84 DNA barcodes), *Bistrispinaria* (1 species, 1 DNA barcodes), *Capparimyia* (4 species, 9 DNA barcodes), *Carpophthoromyia* (5 species, 7 DNA barcodes), *Ceratitis* (53 species, 276 DNA barcodes), *Clinotaenia* (2 species, 2 DNA barcodes), *Dacus* (60 species, 187 DNA barcodes), *Neoceratitis* (1 species, 1 DNA barcode), *Perilampsis* (4 species, 7 DNA barcodes), *Trirhithrum* (14 species, 28 DNA barcodes). The largest part of the vouchers (95.1%) in this library was collected in 30 countries of the African continent (89.5%) or in adjacent islands and archipelagos (Canary Islands, Comoros, La Réunion, Madagascar, Mauritius, Seychelles, 5.6%). The remaining specimens (4.8%) were represented by invasive frugivorous pests collected in Greece, Italy, Spain, Israel, United Arab Emirates, Yemen, India, Indonesia, Pakistan, Philippines and Brazil and nine specimens (1.7%) were of unknown origin (see Data S1). Thirty-three of the species represented in the library are of relevant agricultural importance in Africa [22], [23]. The remaining 120 taxa are currently not considered of economical relevance (Data S1). The library also comprised 85% of all taxa regularly encountered in para-pheromone traps during surveys in different parts of the African continent (http://data.gbif.org/species/13143057). In addition to the library, the 188 interceptions were represented by 49 tephritid species of 7 genera: *Bactrocera* (4 species, 53 queries), *Capparimyia* (1 species, 1 query), *Carpophthoromyia* (5 species, 11 queries), *Ceratitis* (13 species, 36 queries), *Dacus* (19 species, 77 queries), *Perilampsis* (2 species, 2 queries) and *Trirhithrum* (5 species, 8 queries). Five economically important species contributed to 53.2% of the specimens from interceptions. Overall, 68.6% of interceptions belonged to 17 economically important species (Data S1).

**Figure 2:**
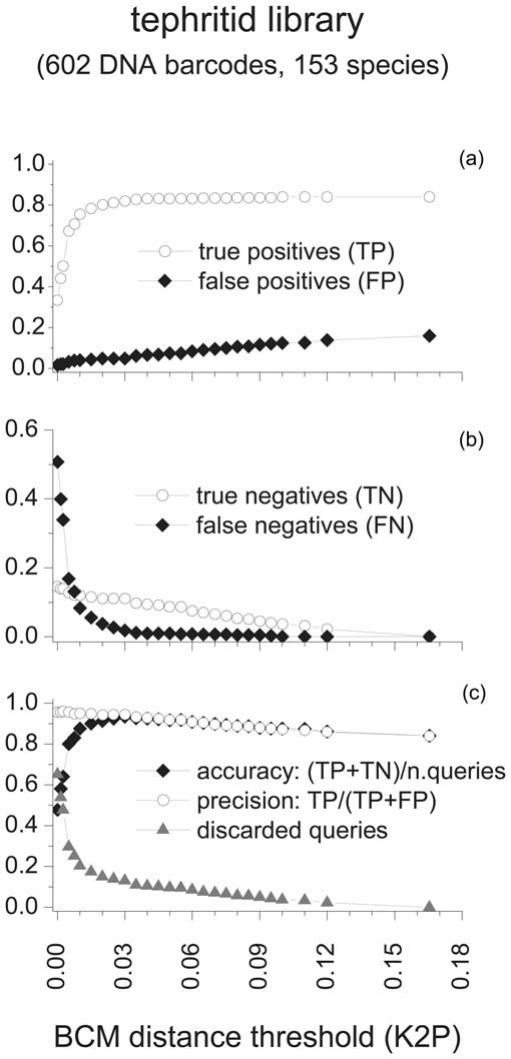
a, b, c: Tephritid Best Close Match (BCM) identification. Proportions of True Positives (TP), False Positives (FP), True Negatives (TN) and False Negatives (FN) at 30 distance thresholds ranging from K2P = 0.165 to K2P = 0.000. For each distance threshold percentages of queries discarded, accuracy ((TP+TN)/total number of queries) and precision (TP/number of not discarded queries) were calculated.

**Figure 3:**
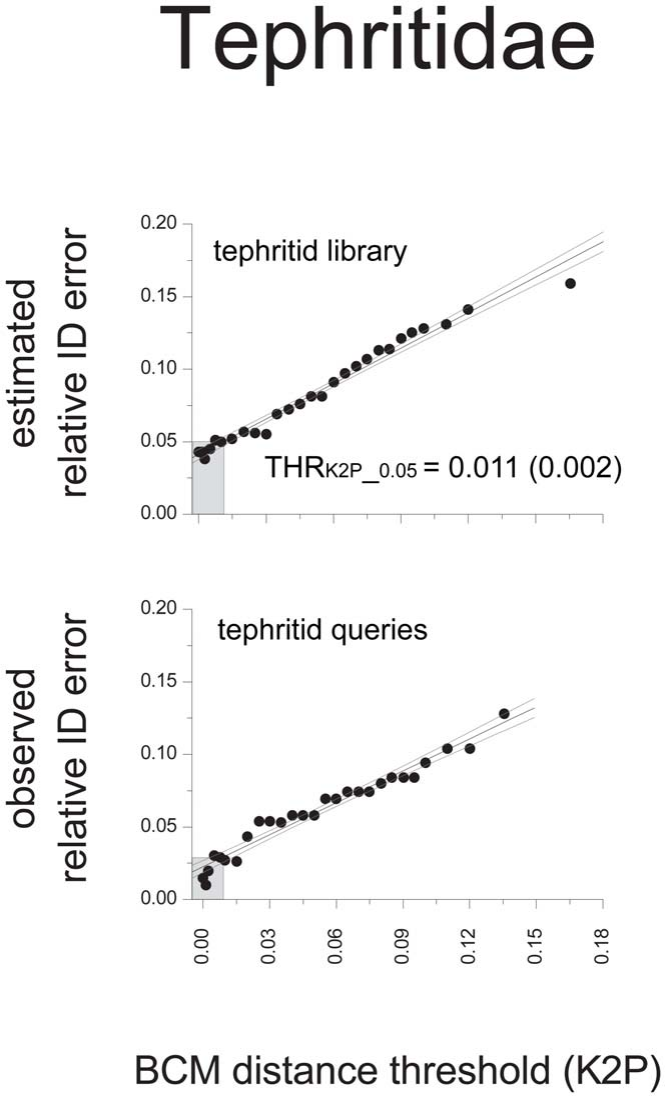
Relative ID errors at 30 arbitrary distance thresholds. Relative ID errors (FP/n. of not discarded queries) obtained from a library of 602 tephritid DNA barcodes. Linear fitting (95% confidence intervals are indicated) was used to infer an *ad hoc* distance threshold corresponding to a relative ID error <0.05 (THR_K2P_0.05_). Error estimates were verified through the DNA barcoding identification of an independent set of 188 tephritid specimens. The grey area represents the relative ID error (estimated and observed) corresponding to THR_K2P_0.05_.

The distribution of pairwise K2P distances in the tephritid library showed that 95% of all the intraspecific distances were in the interval 0.00–7.98%, while 95% of the mean interspecific, congeneric distances were in the interval 6.23–13.55% (Data S2). There was no well-defined barcoding gap as 6.31% of all pairwise comparisons were shared between the 95% percentiles of the intra- and congeneric interspecific K2P distance distributions (*i.e.* fell in the interval 6.23%<K2P<7.98%). BCM simulations in the tephritid library were strongly affected by the K2P distance threshold implemented (Fig. 2a, b, c). The proportion of TP was always markedly higher than the proportion of FP. The proportions of TP and FP decreased as the THR_K2P_ approached 0.00. Yet, while the proportion of FP decreased gradually, the proportion of TP showed a more abrupt decrease for THR_K2P_ ranging from 0.015 to 0.00 (Fig. 2a). The proportions of FN and TN increased the more the distance thresholds approached 0.00 (Fig. 2b). Moving the THR_K2P_ threshold toward 0.00 produced a rapid increase of the proportion of discarded queries (up to 0.651). Accuracy, slowly increased up to 0.934 (at THR_K2P_ = 0.03), then it rapidly decreased reaching a minimum for THR_K2P_ = 0.00. Conversely, precision was positively affected by the use of more restrictive distance thresholds and it gradually increased until a maximum of 0.957 for THR_K2P_ = 0.00 (Fig. 2c). When passing from no threshold (BM) to the most restrictive threshold value (THR_K2P_ = 0.00) precision increased with 11.7%. Overall and relative ID errors showed opposite trends compared to accuracy and precision. Overall ID errors rapidly increased at THR_K2P_ ranging from 0.003 to 0.00 while the relative ID errors gradually decreased for THR_K2P_ approaching to 0.00 (Fig. 3). Linear regression showed that in the tephritid library the K2P distance value corresponding to a relative ID error of 0.05 was THR_K2P___0.05_ = 0.011 (+/−0.002).

The BM criterion allowed a correct identification of 87.2% of the 188 intercepted specimens (Data S3). When THR_K2P_0.05_ was used for the BCM identification of these specimens, the proportion of discarded queries was 0.191 and the proportions of TP, FP, TN and FN were 0.787, 0.021, 0.106 and 0.085, respectively. Among interceptions, THR_K2P_0.05_ produced an overall ID error = 0.106 (range 0.096–0.144 considering the 95% confidence intervals of the threshold estimate) and a relative ID error = 0.026 (range 0.026–0.028 considering the 95% confidence intervals). This resulted in 89.4% of all queries and 97.4% of the not discarded queries being correctly identified (Data S3).

## Discussion

One of the main constraints of DNA barcoding is the difficulty of assembling reference libraries that comprehensively represent the taxonomic diversity of the group to be identified. These limits are evident in insects, where the lack of reference DNA barcodes for approximately 90% of the described species (see http://www.boldsystems.org/views/taxbrowser.php?taxid=82) and for an even higher proportion when considering the estimated number of undescribed taxa [16] implies that insect DNA barcoding may be heavily biased by the misidentification of queries that are not represented in a library [15]. Hence, DNA barcoding, while being considered the future of DNA taxonomy [24], still cannot be reliably used in most real life situations.

Relationships between BCM distance thresholds and variation in identification success were consistent when using large libraries of DNA barcodes from three different insect orders (Data S4, S5, S6). In all simulations it was possible to estimate a distance threshold so that the relative ID error was <0.05. All simulations with the highest taxon coverage (100%) resulted in a less restrictive THR_K2P_0.05_ compared to the lowest taxon coverage (25%). However, only in Hymenoptera we did observe a consistent trend of less restrictive THR_K2P_0.05_ with higher taxon coverages. Variations in the proportion of relative ID errors were less abrupt in libraries with higher taxon coverage. This suggests that libraries properly representing the taxonomic diversity of a particular taxonomic group could better tolerate variations in THR_K2P_0.05_ estimates (as large changes in THR_K2P_0.05_ will produce relatively limited changes in the relative ID error). On the other hand, lower taxon coverage will result in steeper slopes of the linear regressions (so that small changes in THR_K2P_0.05_ will produce large changes in the relative ID error).

In practice, it is very difficult to quantify the effects of low taxon coverage on DNA barcoding identification. When using the BM criterion, a query not represented in the library is always assigned to its closest match leading to an erroneous FP identification. Yet, when using a K2P distance threshold (BCM criterion), misidentifications are distributed between FP and FN. While the proportion of FP is, amongst others, related to levels of taxon coverage, the proportion of FN is not. In fact when using the BCM criterion a query not represented in the library can only be a FP or a TN. A working strategy proposed by Virgilio *et al.* [15] was to use BM identification to rule out the possibility that a query belongs, for example, to a group of pest species (insofar these latter are generally well represented in libraries). This approach would allow the effects of poor taxon coverage to be circumvented because a “negative ID” (*i.e.* query x is not pest species y) is only subjected to the erroneous rejection of a query. Of course, this method remains impractical, as it cannot provide information on all positively identified queries.

In the case of frugivorous tephritids, DNA barcoding might help to identify (invasive) African agricultural pests that otherwise can only be identified by a limited number of experts worldwide. Building a regional tephritid barcode library including all African frugivorous pests is the first step to overcome part of these constraints. Yet, this regional library is far from representing the entire taxonomic diversity of the Tephritidae, an insect family including nearly 1,000 species in Africa. Hence, while the regional tephritid barcode library could be useful for interception purposes (*i.e.* for identifying known African agricultural pests), it should not be considered as an identification tool for all African tephritids.

As in many other taxa, tephritids include a relatively small proportion of very common species and a relatively large number of rare species [25]. Tephritid agricultural pests are common and widespread, usually intercepted through commercially available pheromones [26]. By using a library biased towards agricultural pests we accept that many rare and/or economically unimportant species may be misidentified. Even if we expected a limited error related to the misidentification of rare species (for these taxa being uncommon by definition), the BM identification of tephritid interceptions (no threshold considered) had a low ID success, with only 87.2% of specimens correctly identified. Most of the mismatches between morphological and BM identifications were related to the lack of a query conspecific in the library (70.8% of all misidentified specimens, 61.1% of all misidentified species, see Data S3). Other misidentifications involved (1) queries of species belonging to species complexes or to species groups with well-known limited interspecific divergence, *e.g. Ceratitis anonae*, *C. capitata*, (8.3% of queries, see also [27]), (2) queries of species with high intraspecific divergence and in need of taxonomic revision, *e.g. Dacus humeralis*, (4.2% of queries) or (3) various laboratory related issues (*e.g.* contamination or mislabeling, 4.2% of queries). Introducing a distance threshold greatly increased levels of precision in the identification of queries. Higher precision was obtained with more restrictive thresholds and the highest precision value in the tephritid library was obtained when only identical matches between queries and reference DNA barcodes were considered (THR_K2P_ = 0.00, correct ID of 95.7% of not discarded queries). Of course, higher precision also resulted in lower accuracy due to the higher proportions of queries discarded (65% of queries discarded at THR_K2P_ = 0.00). In an effort to balance the relatively poor performance of BM identification and the impractical use of too restrictive distance thresholds, we adopted a pragmatic approach based on an estimated 5% probability of misidentification. Considering that in tephritids this value cannot be reached through BM identification (as BM brings to the misidentification of 12.8% of queries) we used a library of DNA barcodes and estimated a theoretical BCM distance threshold producing a relative ID error <0.05. Accordingly, when the estimated distance threshold was used in the BCM identification of 188 tephritid interceptions, it produced a relative ID error of 2.6–2.8% (considering the 95% confidence intervals of the threshold estimate). Remarkably, using the threshold allowed (1) 83.3% of queries misidentified through the BM criterion to be discarded and (2) the proportion of misidentified queries due to the lack of a conspecific DNA barcode in the reference library (now representing only 25% of misidentified queries) to be markedly reduced. Still, the proportion of queries not identified (*i.e.* discarded) was relatively low (19.1%, range 18.1–22.9%).

In this work we propose a method to estimate *ad hoc* distance thresholds to be used in the BCM identification of species. This method (Data S7) represents a compromise between the sometimes-poor performance of DNA barcoding [14], [15] and the need for general protocols to be used in DNA barcoding [28]. An *ad hoc* threshold should be used as a cut-off mark defining whether (1) we can proceed and identify the query with a known estimated error probability (*e.g.* 5%) or (2) whether we should discard the query and consider alternative/complementary identification methods. This pragmatic identification tool based on DNA barcoding still heavily relies on “traditional” morphological taxonomy which, on the one hand is involved in the certification of vouchers used to assemble the library and, on the other hand, in the identification of discarded queries whose K2P distances are above the threshold.

## Supporting Information

Data S1 Morphological identification, sampling locations and DNA barcode sequences of 602 reference tephritid specimens (tephritid reference library, sheet 1) and 188 intercepted tephritids (tephritid interceptions, sheet 2). The economic status of speces included in the two datasets is summarised in sheets 3 and 4.
(XLS)

Data S2 Diptera. Best Close Match (BCM) identification. Proportions of True Positives (TP), False Positives (FP), True Negatives (TN) and False Negatives (FN) at 30 distance thresholds. For each distance threshold percentages of a) queries discarded, b) accuracy ((TP+TN)/total n. of queries) and c) precision (TP/n. of not discarded queries) were calculated.
(EPS)

Data S3 Hymenoptera. Best Close Match (BCM) identification. Proportions of True Positives (TP), False Positives (FP), True Negatives (TN) and False Negatives (FN) at 30 distance thresholds. For each distance threshold percentages of a) queries discarded, b) accuracy ((TP+TN)/total n. of queries) and c) precision (TP/n. of not discarded queries) were calculated.
(EPS)

Data S4 Lepidotera. Best Close Match (BCM) identification. Proportions of True Positives (TP), False Positives (FP), True Negatives (TN) and False Negatives (FN) at 30 distance thresholds. For each distance threshold percentages of a) queries discarded, b) accuracy ((TP+TN)/total n. of queries) and c) precision (TP/n. of not discarded queries) were calculated.
(EPS)

Data S5 Distributions of interspecific (grey squares) and intraspecific (white circles) pairwise K2P distances in a library of 602 tephritid DNA barcodes. In grey: overlap between the 95% percentiles of intra- and interspecific distributions (6.23%<K2P<7.98%)
(EPS)

Data S6 DNA barcoding identification of 188 intercepted tephritid specimens using a reference library of 622 tephritid DNA barcodes (see Data S1). For each specimen, best DNA barcode match, genetic distance, number of base pair (bp) differences, bp overlapping query-best match, outcome of best match identification (ID) and of best close match ID (with a distance threshold corresponding to THR_K2P_0.05_ = 0.011) are indicated. Specimens correctly identified according to the best match and best close match criteria are highlighted in grey, identification errors are in yellow. TP: true positive, FP: false positive, TN: true negative, FN: false negative. Species (a) not represented in the reference library, (b) belonging to species complexes or to species groups with known low interspecific divergence and (c) with high intraspecific divergence and in need of taxonomic revision are indicated.
(XLS)

Data S7 *Ad hoc* distance thresholds for Best Close Match (BCM) identification.
(DOC)
