# Supplementary material for: Identifying Insects with Incomplete DNA Barcode Libraries, African Fruit Flies (Diptera: Tephritidae) as a Test Case
Source: PLoS One. 2012 Feb 16;7(2):e31581. doi: 10.1371/journal.pone.0031581 (PMC3281081; doi:10.1371/journal.pone.0031581)
Supplement: Data S7 — Ad hoc distance thresholds for Best Close Match (BCM) identification. (DOC) [file pone.0031581.s007.doc]

Data S7

*Ad hoc* distance thresholds for Best Close Match (BCM) identification:

1. build a library of DNA barcodes for the particular taxonomic group of interest
2. use each DNA barcode as a query against all the other DNA barcodes of the library and calculate query-best match distances (*e.g.* using SpeciesIdentifier, Meier *et al.* 2006)
3. establish a number of arbitrary distance thresholds (*e.g.* 30) from the largest observed pairwise distance to K2P = 0.00
4. for each distance threshold quantify the precision levels (TP/(TP+FP)) and infer the relative ID error (=1-precision).
5. perform a regression analysis and estimate the distance threshold (and confidence intervals) corresponding to a relative ID error < 0.05. If this *ad hoc* threshold THRK2P_0.05 < 0 then a precision > 0.95 cannot be reached and the library should be considered as unreliable.
6. use the estimated THRK2P_0.05 for BCM identification
7. discard queries above threshold and identify them with alternative morphological/molecular methods
8. new properly identified queries can be added to the library and THRK2P_0.05 recalculated from a more complete reference library
